# Supplementary material for: Nuclear spin assisted quantum tunnelling of magnetic monopoles in spin ice
Source: Nat Commun. 2019 Apr 3;10:1509. doi: 10.1038/s41467-019-09323-6 (PMC6447640; doi:10.1038/s41467-019-09323-6)
Supplement: Supplementary file 1 — Supplementary Information [file 41467_2019_9323_MOESM1_ESM.pdf]

Supplementary Information

Nuclear spin assisted quantum tunnelling of magnetic monopoles in spin ice

C. Paulsen,[1*] S. R. Giblin,[2] E. Lhotel,[1] D. Prabhakaran,[3]

K. Matsuhira,[4], G. Balakrishnan.[5] S. T. Bramwell.[6]

[1]Institut Néel, C.N.R.S - Université Grenoble Alpes, 38042 Grenoble, France.

[2]School of Physics and Astronomy, Cardiff University, Cardiff, CF24 3AA, United Kingdom.

[3]Clarendon Laboratory, Physics Department, Oxford University,

Oxford, OX1 3PU, United Kingdom.

[4]Kyushu Institute of Technology, Kitakyushu 804-8550, Japan.

[5]Department of Physics, University of Warwick, Coventry, CV4 7AL, United Kingdom.

[6]London Centre for Nanotechnology and Department of Physics and Astronomy,

University College London, 17-19 Gordon Street, London, WC1H 0AJ, United Kingdom.

[*] E-mail: carley.paulsen@grenoble.cnrs.fr

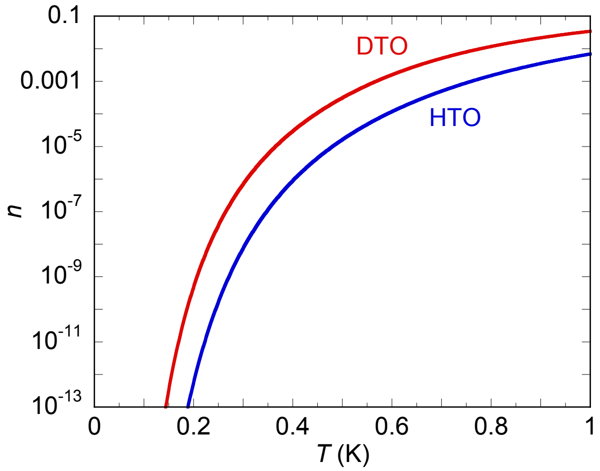

Supplementary Figure 1: **Density of single-charge monopoles (equilibrium number per diamond lattice site in zero field) versus temperature**, calculated by the Debye-Hückel theory of Kaiser et al.[1]. The analytic calculation is very accurate for the monopole model of spin ice: it includes both single and double-charge monopoles, but only the single-charge monopoles are relevant at the temperatures we study. The density of 'flippable' spins per spin site is 3/2 times the monopole density.

Supplementary Note 1: Experimental details

Creating a large density of monopoles using the avalanche quench protocol in $Ho_2Ti_2O_7$. We have previously described the avalanche quench protocol (AQP) in detail for $Dy_2Ti_2O_7$ (see Ref. 2 and its Supplementary Information). From magnetisation measurements recorded during and after the AQP, we inferred that samples of $Dy_2Ti_2O_7$ heat systematically to temperatures above 900 mK, even though the reference thermometer on the sample holder only registered a small jump[3].

However from the onset, it was clear that $Ho_2Ti_2O_7$ was different. For example, when performing measurements where the field is ramped at a steady rate, the magnetic avalanches of $Ho_2Ti_2O_7$ never reach the 900 mK equilibrium value as seen in Fig. 4c. Sometimes depending on previous measurements, the AQP worked very poorly, or did not seem to work at all.

This was the motivation for measuring the sample temperature directly by mounting a thermometer on the samples during some of the runs.

Temperature measurements during the AQP. We attempted direct temperature measurements with 4 different thermometers; 2 homemade RuO_2 resistance thermometers (filed down to reduce mass with wires attached with silver epoxy), a Cernox 1010-SD thermometer with platinum leads, and a bare-chip Cernox 1010-BC, both from LakeShore Cryogenics. All had short comings but in the end most of the measurements shown here were made using the bare chip thermometer. This was the

lightest of the four, but had a small but noticeable magnetoresistance that we have corrected for. A constant current source delivered 10 nA, and the voltage was measured with a Stanford Instruments model 830 lock-in amplifier running at 1100 Hz. This setup was a compromise, the measurements of the temperature were fast, but prone to some drift and noise.

During normal measurements samples are sandwiched between two long narrow pieces of Cu that are anchored to the mixing chamber of a miniature dilution refrigerator. The samples are glued in place, then teflon tape is tightly wrapped around the Cu strips, clamping the samples to the Cu. For measurements with the thermometer glued to the sample, only one Cu strip was used, the second was suspended away from the thermometer, as shown in Supplementary Figure 2.

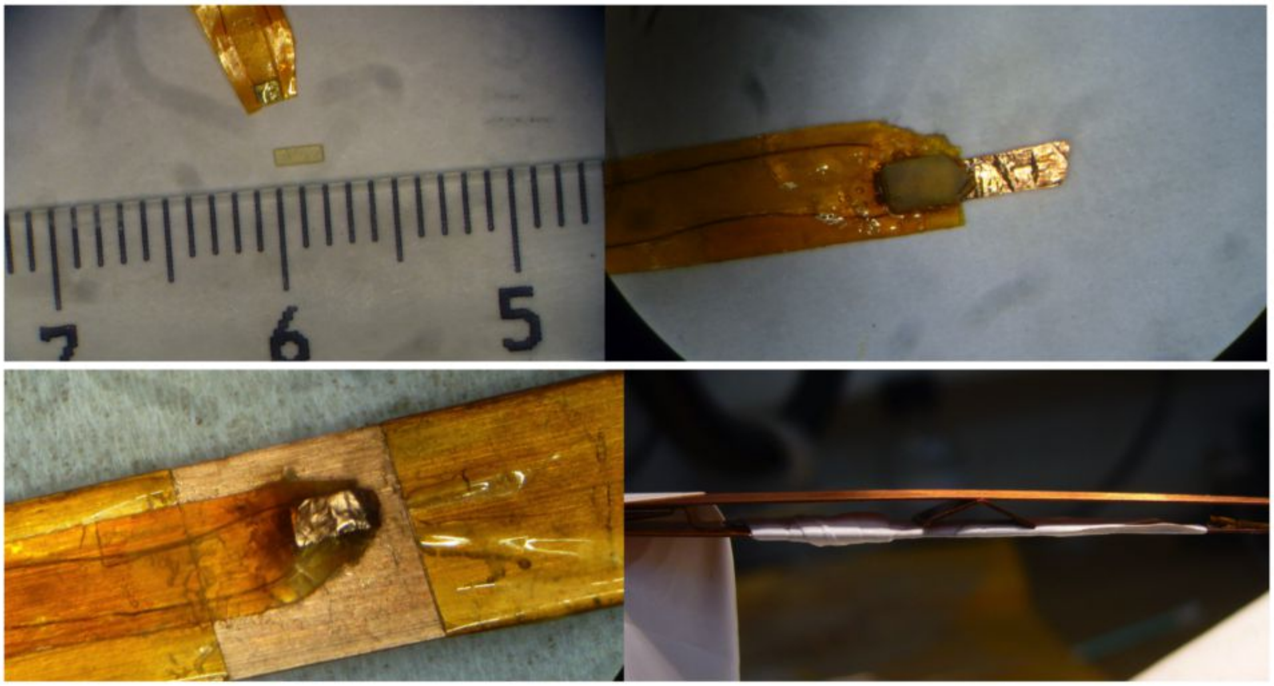

Supplementary Figure 2: **Pictures of the sample mounting**. Upper left: Sample of $Dy_2Ti_2O_7$ and Cernox bare chip resistor with leads protected by kapton tape. Upper right: $Ho_2Ti_2O_7$ glued onto the bare chip resistor with thin Cu foil protruding. Lower left: the sample + thermometer have been turned over, and the sample surface has been glued onto the Cu sample holder using GE varnish. The Cu foil has been folded back to cover the upper half of the sample and bare chip resistor. Lower right: standoffs hold upper part of sample holder away, teflon tape has been wrapped around the sample and thermometer. The thermometer is isolated from the Cu sample holder by the sample.

Supplementary Note 2: Results

As already discussed, cooling samples that contain Ho can be problematic, and this is also true for heating samples with the AQP when the starting temperature was well below 200 mK. This is shown in Supplementary Figure 3 for a series of AQP taken on $Ho_2Ti_2O_7$ when the sample was first cooled to 65 mK after waiting 4 hours. Point (a) is the beginning of sequence when we applied a field of -0.3 T on the sample followed by a wait period of 180 s. For $Dy_2Ti_2O_7$, already a large > 1 K spike in temperature would be seen at (a) as the sample rapidly magnetizes in the field, but for $Ho_2Ti_2O_7$ only a small jump of about 0.3 K was recorded. The jump in temperature at the first AQP is also small, only reaching about 0.6 K, compared to > 1.4 K for $Dy_2Ti_2O_7$ under similar conditions. At (c) we begin a second AQP, again setting $\mu_0 H = -0.3$ T, and waiting 180 s. But this time the jump in temperature reaches nearly 0.8 K, and the second AQP at (d) shows that this time, the sample has warmed > 1.3 K.

During the AQP, heat from the flipping of the electronic spins is absorbed by the sample. But because of the large heat capacity of the Ho nuclei, much of the energy is absorbed by the nuclear spin bath, raising its temperature but resulting in a small overall jump in sample temperature. However for the second AQP, the starting sample temperature is now greater, nearly 0.16 K, and this is enough to heat the spins above one Kelvin.

Thus $Ho_2Ti_2O_7$ measurements, were systematically made with 2 or 3 AQP in succession in order to ensure the sample is warmed above 1 K.

Note that the need for several AQP also suggests that at low temperature, well below 300 mK, the nuclear spins begin to freeze out, and anti-align with their respective electronic spin, thus 2 in – 2 out for the electronic spin becomes 2 out – 2 in for its nuclear counterpart.

Another nagging problem was that the samples of $Ho_2Ti_2O_7$ did not reach $M = 0$ after the AQP. The origin of this is not clear, but our data suggests that $Ho_2Ti_2O_7$ cools too fast. When the field is switched off, the applied field H goes to zero before the sample even starts to change its magnetisation. The sample then feels the internal field $H_{\text{internal}} = -D \cdot M$ where D is the demagnetisation factor and avalanches against this. As the magnetisation decreases, H_{internal} also decreases, the sample heats, but then cools so rapidly that the magnetisation gets 'stuck' at a small positive value of the order 1 emu/g. For convenience, the solution was to add a small overshoot for the field of about -0.004 T for 1 s, then switch back to $H = 0$. This resulted in a starting M closest to zero. Note that measurements without the overshoot gave the same results, but with an offset. This ultra rapid cooling may also explain why the avalanches shown in Fig. 4c for $Ho_2Ti_2O_7$ (while ramping of the field) fall below the equilibrium value expected for 900 mK, in contrast to $Dy_2Ti_2O_7$.

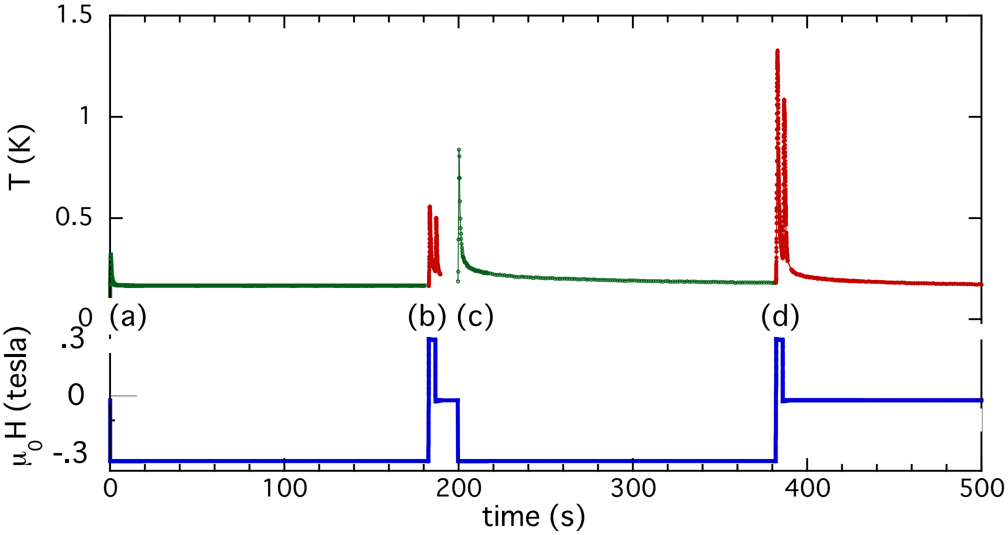

Supplementary Figure 3: The applied field and the sample temperature for $Ho_2Ti_2O_7$ as a function of running time for a double AQP. The sample was cooled for 4 hours to base temperature of approximately 65 mK. (a) At $t = 0$, the field was changed from 0 to -0.3 T. A relatively weak jump in the temperature can be seen. (b) At $t = 180$ s, the first AQP is performed: the field goes from -0.3 to $+0.3$ T, then after 4 s from $+0.3$ T to zero. The temperature on the sample reaches about 0.55 K, not sufficient to randomize the spins. (c) At $t = 200$ s the field is again put at -0.3 T in preparation for the next AQP. This time the jump in sample temperature is larger, but still less than required. (d) At $t = 380$ s the second AQP takes place, warming the sample above 1 K.

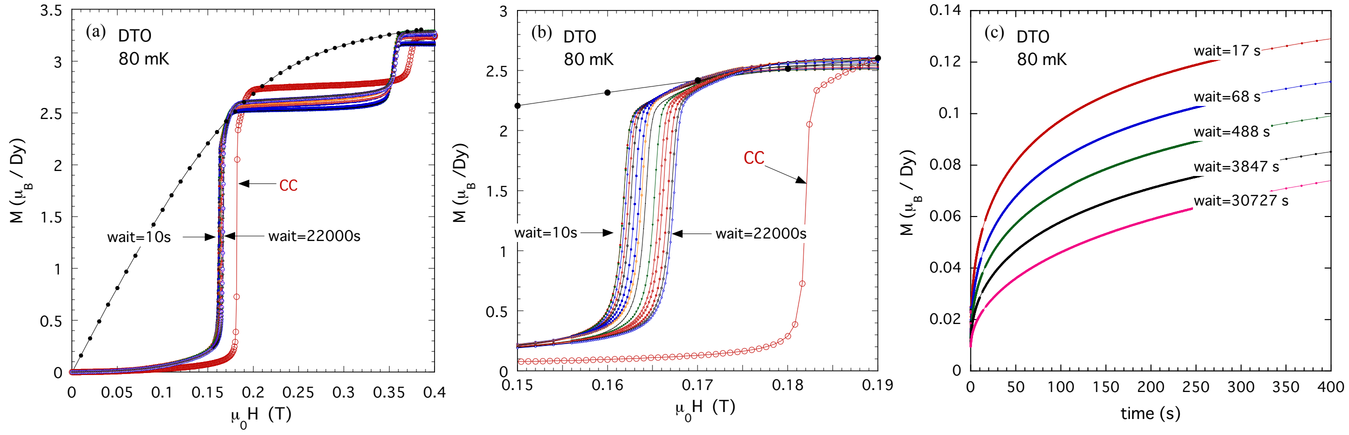

Supplementary Figure 4: Plots for natural $Dy_2Ti_2O_7$ corresponding to Figs. 3 and 4 of the main manuscript. (a) Avalanches of the magnetisation recorded while the field was ramped at 0.02 T/s for $^{nat}Dy_2Ti_2O_7$ (DTO). The samples were first prepared using the AQP and then followed by various wait times except for the curve marked 'ZFC', where the sample was first prepared using the conventional zero field cooled (CC) protocol (red circles). Also shown is the equilibrium M vs $\mu_0 H$ taken at 900 mK (solid black dots). (b) Magnification, showing the spread in avalanche fields as a function of the wait time, and the ZFC far outside the pack. (c) The effect of wait time on the relaxation of the magnetisation M vs time for $^{nat}Dy_2Ti_2O_7$ measured at 80 mK. The samples were again first prepared using the AQP. After the specified wait periods, a field of 0.08 T was applied and the magnetisation as a function of time was recorded.

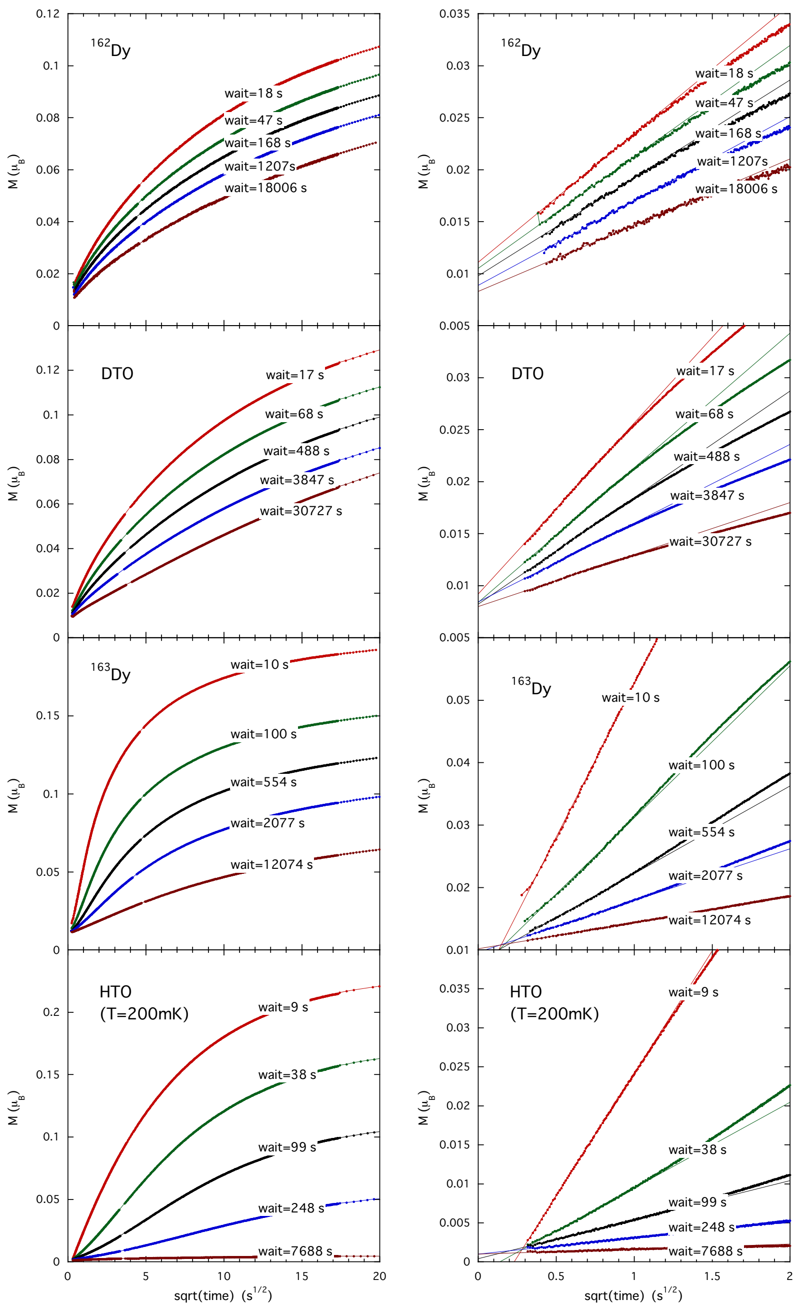

Supplementary Figure 5: **Time dependence of the magnetisation at short times**. The same data of Fig. 3 in the main text as well as data for the $^{nat}Dy_2Ti_2O_7$ sample plotted against $\sqrt{time}$. An important prediction from Prokofev and Stamp[4] was that the initial relaxation of the magnetisation should follow a square root time dependence. This worked well for the SMM Fe_8 up to 1000 s or more. The situation for spin ice is quite different, the right hand side of the figure shows the data can at best be fitted over a very restricted range in time only up to 1 s.

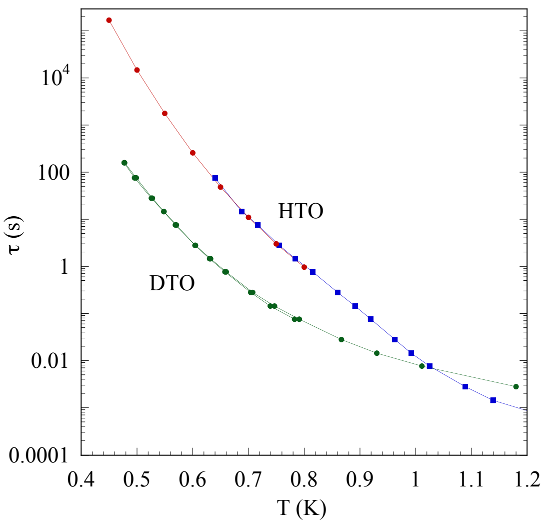

Supplementary Figure 6: Experimental values of relaxation time τ from susceptibility and magnetisation measurements. Relaxation time τ vs temperature for $Ho_2Ti_2O_7$ and $^{nat}Dy_2Ti_2O_7$. The green and blue data points (τ less than 100 s) were taken from the peaks in the imaginary susceptibility. The red points for $Ho_2Ti_2O_7$ come from analyzing dc relaxation (all raw data was first corrected for demagnetisation effects). The slope $dT/d\tau$ defining the equilibrium cooling rate shown in Fig. 2 are taken from fits to these curves.

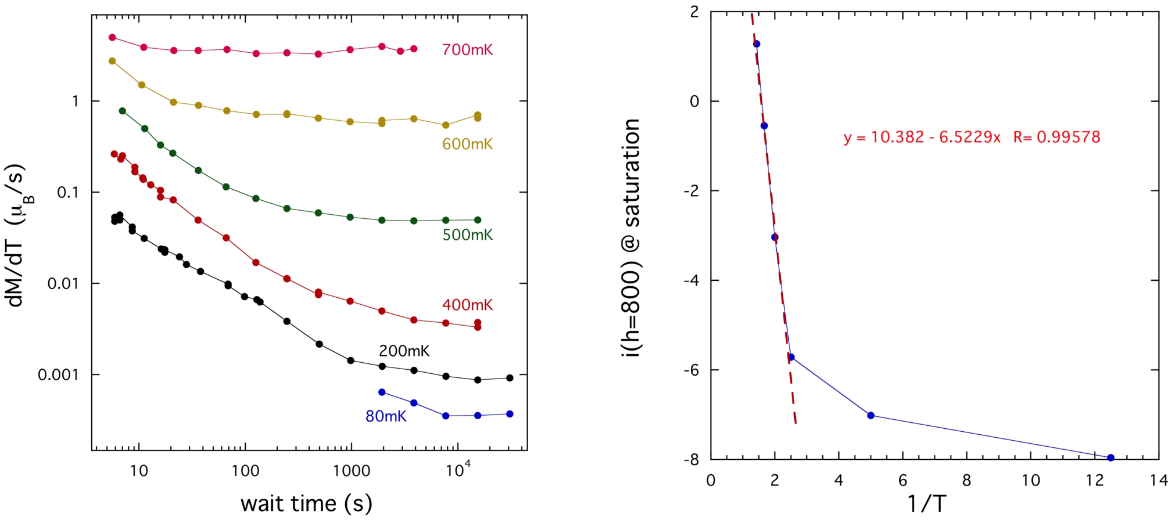

Supplementary Figure 7: Effect of monopole current $J_m = dM/dt$ on wait time for $Ho_2Ti_2O_7$ left: Monopole current $J_m = dM/dt$ obtained from the relaxation of the magnetisation of $Ho_2Ti_2O_7$ measured after different waiting times, and measured at 800 Oe. J_m is obtained by extrapolating the derivative of the magnetisation with respect to time at $t = 0$ (See Ref. 2 for the detailed procedure). right: J_m vs $1/T$ obtained from the saturation value of the left figure, i.e. when the current value does not depend anymore on the waiting time.

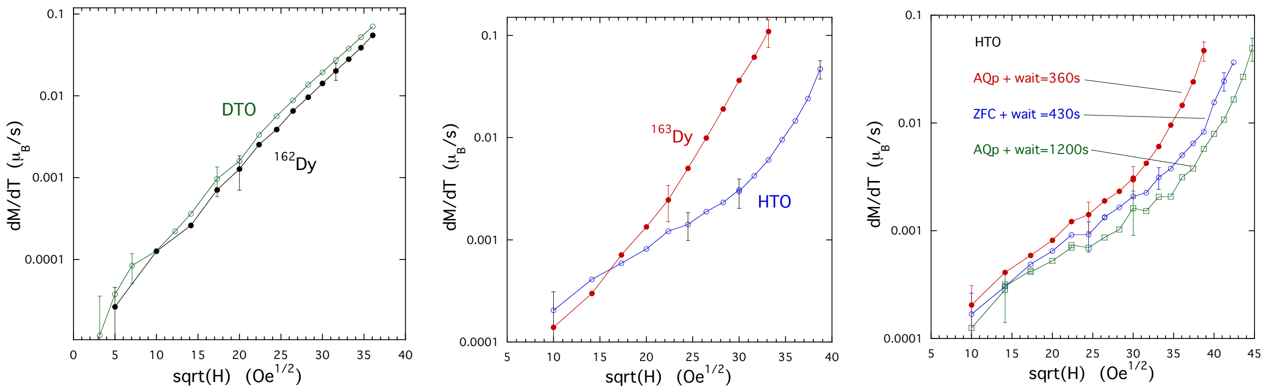

Supplementary Figure 8: **Effect of the nuclear spins on the monopole current**. Monopole current $J_m = dM/dt$ vs $\sqrt{H}$ determined for $^{\rm nat}$Dy and ^{162} $Dy_2Ti_2O_7$ samples (left), ^{163} $Dy_2Ti_2O_7$ and $Ho_2Ti_2O_7$ samples (middle), and in different cooling conditions for the $Ho_2Ti_2O_7$ sample. J_m is obtained by extrapolating the derivative of the magnetisation with respect to time at $t = 0$ (See Ref. 2 for the detailed procedure). Natural and ^{162}Dy (no nuclear spin) $Dy_2Ti_2O_7$ follows the $\sqrt{H}$ behavior expected for magnetic monopoles interacting through the Coulomb force, $^{163}Dy_2Ti_2O_7$ and $Ho_2Ti_2O_7$ samples do not, whatever the cooling process and so the initial density of monopoles. This result shows, as suggested in the main text, that the idealised emergent chemical kinetics of monopole theory does not apply in $^{163}Dy_2Ti_2O_7$ and $Ho_2Ti_2O_7$, where the dynamics is strongly affected by the nuclear spin effects, because the hyperfine splitting energies are of a similar order to the Coulomb energies.

Supplementary Note 3: Different Samples and Measuring Directions

During the course of this study, 10 different samples were measured with some samples measured along multiple axis. The mass of the samples ranged between 3 to 40mg, and they had various shapes. No corrections for demagnetization effects have been taken into account for the results presented in the main text. This is because for most of the measurements shown in the main text, the sample was far from equilibrium, and the magnetization was very small, and thus the demagnetizing field -NM was small. Nevertheless the sample shape and field direction do effect the observed relaxation curves and the avalanche fields.

In this section we show that although there are some variations between samples, between cooling runs, and for different directions, these differences do not change the main conclusion of the paper; the demonstration that nuclear assisted quantum tunneling is operative regardless of field direction.

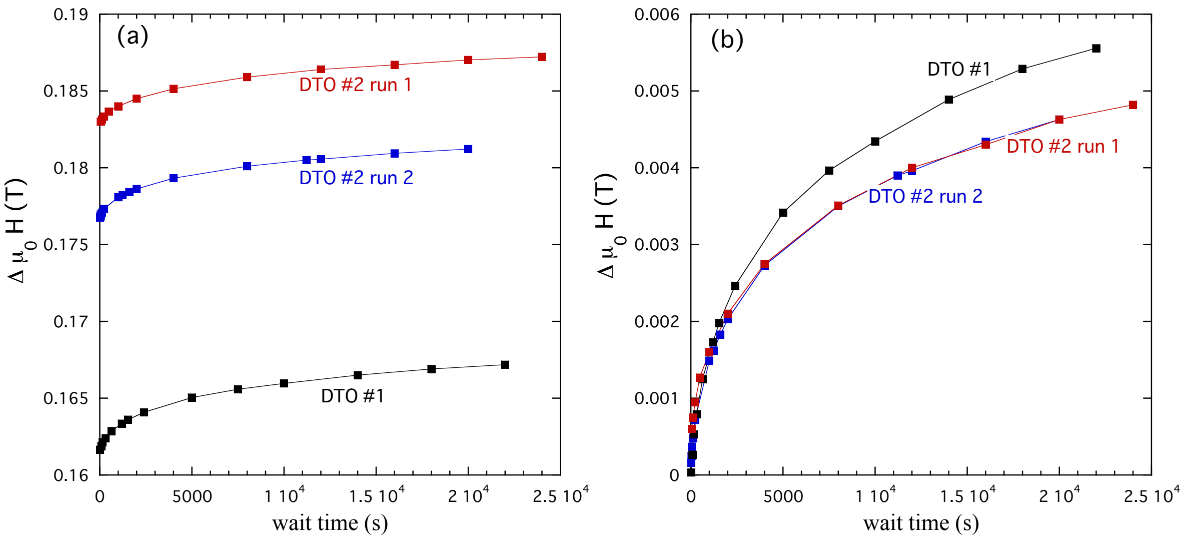

Supplementary Figure 9: The effect of wait time t_{w} on the magneto-thermal avalanches for two different samples of natural DTO, and for two different runs (a) shows the value of the avalanche field $H_{\mathrm{ava}}(t_{\mathrm{w}})$, defined as the field where the magnetization crosses 1 μ_{B} per rare earth ion. (b) is a plot of the difference in avalanche field $\Delta H_{\mathrm{ava}} = H_{\mathrm{ava}}(t_{\mathrm{w}}) - H_{\mathrm{ava}}(t_{\mathrm{w}} = minimum)$.

Supplementary Figure 9(a) shows the effect of t_{w} on the magneto-thermal avalanches for two different samples of natural DTO, and for two different runs. Sample 1 (also shown in the main text) was rectangular shaped parallelepiped and sample 2 was a square thin platelet shaped sample. The measurements shown in the figures were taken with the field along the [111] axis for both samples. The left panel shows the value of the avalanche field $H_{\mathrm{ava}}(t_{\mathrm{w}})$, defined as the field where the magnetization crosses 1 μ_{B} per rare earth ion. The curves are clearly offset from one another, even the two curves taken on the same sample, but during different runs. The initial position of the avalanche field is

very sensitive to thermal contact with the sample holder. For sample 2 run 1, the thermal contact was made using two Cu bands with the sample sandwiched between the two. For sample 2 run 2 only one Cu band was used, thus the thermal contact was worse. The better thermalized sample has a higher avalanche field, because leading up to the avalanche heat could be more efficiently evacuated from the sample. Supplementary Figure 9(b) is a plot of the difference in avalanche field $\Delta H_{\mathrm{ava}} = H_{\mathrm{ava}}(t_{\mathrm{w}}) - H_{\mathrm{ava}}(t_{\mathrm{w}} = minimum)$. As can be seen, for sample 2, the two runs collapse onto one another, but the shape of the curve for sample 1 is slightly different. A more systematic study needs to be made to understand if this is a shape dependent effect, or sample dependent.

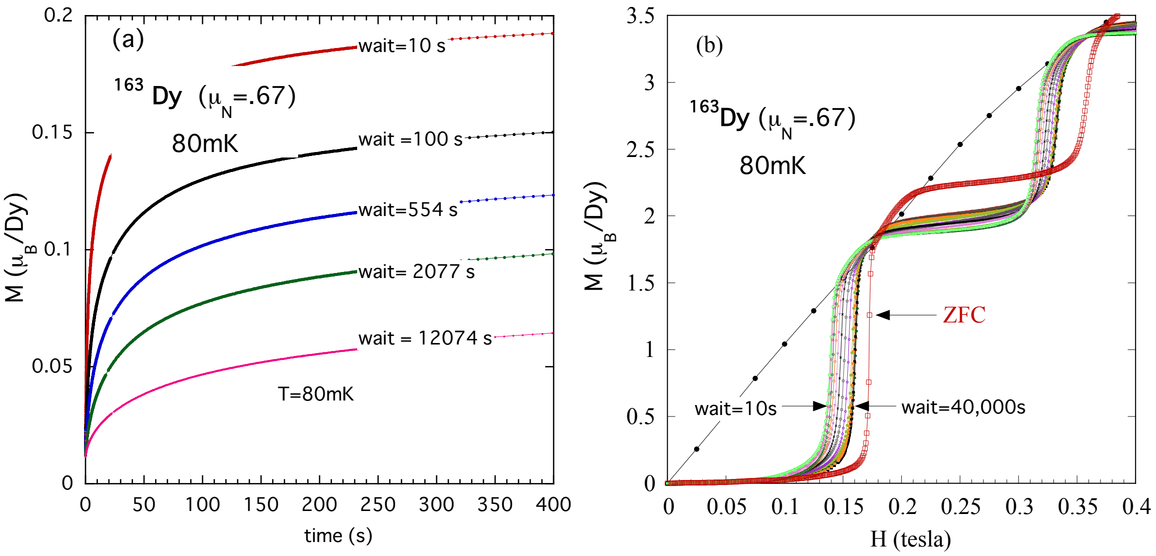

Supplementary Figure 10: **Measurements made on a polycrystalline sample of ^{163}Dy$_2$Ti$_2$O$_7$ (sample 2)** The sample was first prepared using the same avalanche quench protocols (AQP) outlined in the main text and methods section, followed by various waiting times. (a) shows the effects of wait time on the relaxation of the magnetization in a field of 0.8 T at 80mK. (b) shows the effects of wait time on the position of the avalanche field when the field is ramped from 0 to 0.4 T at a constant rate of 0.02 T/s at 80mK. Also shown in the figure is the conventional zero field cooling (CC) curve were the sample was slowly cooled from 900 mK to 80 mK (at 1 mK/s) followed by a 1000 s wait period. For this sample the CC avalanche field is offset to higher fields, and is well outside the distribution of $H_{\mathrm{ava}}(t_{\mathrm{w}})$.

For ^{163}Dy$_2$Ti$_2$O$_7$, two samples were studied. Sample 1 was an odd shaped disk. The [111] direction was perpendicular to the surface of the disk, and resulted in a very large demagnetization factor for this direction. Importantly this resulted in difficulty thermalizing the sample along this direction to our Cu sample holder. This resulted in a much less efficient AQP cooling. We estimate that the sample took about 5 seconds to cool below 500mK, and about 40 seconds to cool below 100mK. This is much slower than the usual AQP as described in Fig. 2 of the main text, but still faster than the CC

method. Sample 1 was measured along the [111] direction (shown in the main text) and perpendicular to the [111] direction. Sample 2 was a polycrystalline sample and the effects of wait time on the relaxation of the magnetization and position of the avalanche field are shown in Supplementary Figure 10 (a) and (b). These data sets are very similar to those presented in the main text in terms of the strength of the effect of wait time for $^{163}Dy_2Ti_2O_7$. (see Figures 3 and 4)

However there are two interesting differences.

Firstly, Supplementary Figure 11 (a) shows the monopole current $J_{\rm m} = dM/dt$ at $t = 0$ vs log wait time for sample 1 [111] and perpendicular to [111] as well as for polycrystalline sample 2. As can be seen in the figure, although the slopes of the three curves are roughly the same, the [111] data fall significantly below the two perpendicular curves. Most likely this is not an intrinsic effect, but comes from the poor thermalization for the [111] sample run: as mentioned above, for this direction after the AQP the sample cooled much slower, therefore the initial monopole density at the beginning of the wait period was much reduced, so the initial monopole current was less, shifting the [111] curve down in the plot. A second difference that can be seen in Supplementary Figure 10 (b) is that the avalanche field for the CC method occurs at much higher fields and is well outside the distribution of curves obtained by the AQP method. The same result was found for sample 1 perpendicular to [111]. This can be contrast to the data shown for the [111] sample in the main text, and again can be explain by the slower cooling for the [111] sample run.

The results for measurements on 3 different samples of HTO are also shown in Supplementary Figure 11. Sample 1 was a needle shaped sample measured along the [111] (long) direction. Sample 2 was also needle shaped and measured along the [001] direction. Sample 3 was a square platelet, and was measure along the [001] and [110] axis. Supplementary Figure 11 (a) shows the monopole current $J_{\rm m} = dM/dt$ at $t = 0$ vs log wait time for sample 1 [111] (also in the main text) compared to sample 2 [110]. The effect of wait time on the currents for these two samples are very similar; the rate at which monopoles recombine is seen to be much faster than that of $^{163}Dy_2Ti_2O_7$, and both seem to saturate at very long wait times. Supplementary Figure 11 (b) are plots of difference in avalanche field $\Delta H_{\rm ava} = H_{\rm ava}(t_{\rm w}) - H_{\rm ava}(t_{\rm w} = minimum)$ against log wait time for the three samples of HTO, as well as two samples of $^{163}Dy_2Ti_2O_7$ and for two samples of $^{\rm nat}Dy_2Ti_2O_7$ (DTO) (same data as shown in Supplementary Figure 9 (b)).

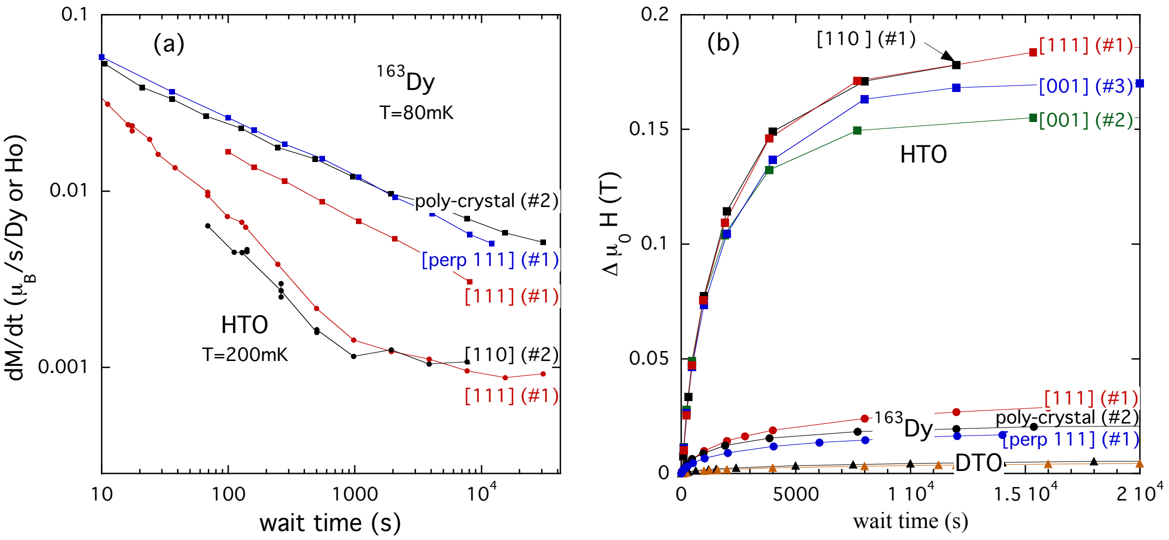

Supplementary Figure 11: **comparison of different samples and different measuring directions for HTO, ^{163}Dy$_2$Ti$_2$O$_7$ and ^{nat}Dy$_2$Ti$_2$O$_7$ (DTO)** (a) the monopole current $J_m = dM/dt$ at $t = 0$ vs log wait time for two samples of ^{163}Dy$_2$Ti$_2$O$_7$ and three samples of HTO. (b) Plot of difference in avalanche field $\Delta H_{ava} = H_{ava}(t_w) - H_{ava}(t_w = minimum)$ against wait time for various directions and various samples of HTO, ^{163}Dy$_2$Ti$_2$O$_7$ and ^{nat}Dy$_2$Ti$_2$O$_7$ (DTO). The top 4 curves in the figure are measurements for 3 different samples of HTO (squares). Sample 1 was a needle shaped sample measured along the [111] (long) direction. Sample 2 was also needle shaped and measured along the [001] direction. Sample 3 was a square platelet, and was measure along the [001] and [110] axis. The middle 3 curves are for two different samples of ^{163}Dy$_2$Ti$_2$O$_7$(solid dots) measured at 80mK. Sample 1 was measured along the [111] direction and perpendicular to the [111] direction, and sample 2 was a poly-crystal. The bottom two curves are for natural DTO (triangles) taken on two different samples along the [111] and [001] directions. (same data as shown in Supplementary Figure 9 (b))

Supplementary References

1. Kaiser, V., Bloxsom, J. A., Bovo, L., Bramwell, S. T., Holdsworth, P. C. W. & Moessner, R. Emergent Electrochemistry in Spin Ice: Debye–Hückel Theory and Beyond. *Phys. Rev. B* **98**, 144413 (2018).

2. Paulsen, C., Giblin, S. R., Lhotel, E., Prabhakaran, D., Balakrishnan, G., Matsuhira, K., and Bramwell S. T. Experimental signature of the attractive Coulomb force between positive and negative magnetic monopoles in spin ice. *Nature Physics* **12**, 661 (2016).

3. Jackson, M. J., Lhotel, E., Giblin, S. R., Bramwell, S. T., Prabhakaran, D., Matsuhira, K., Hiroi, Z., Yu, Q., and Paulsen, C. Dynamic behavior of magnetic avalanches in the spin-ice compound $Dy_2Ti_2O_7$. *Phys. Rev. B* **90**, 064427 (2014).

4. Prokof'ev, N. V. & Stamp, P. C. E. Low-temperature quantum relaxation in a system of magnetic nano molecules. *Phys. Rev. Lett.* **80**, 5794 (1998).
